# Classification of patients with breast cancer according to Nottingham Prognostic Index highlights significant differences in immunohistochemical marker expression

**DOI:** 10.1186/1477-7819-12-243

**Published:** 2014-08-01

**Authors:** Fisnik Kurshumliu, Lumturije Gashi-Luci, Shahin Kadare, Mehdi Alimehmeti, Ugur Gozalan

**Affiliations:** 1 Institute of Anatomic Pathology, University Clinical Center, Medical School, University of Pristina, Pristina, Kosovo; 2 Laboratori Diagnostik Morfopatologjik, Tirana, Albania; 3 Shërbimi i AnatomisëPatologjike, Qendra Spitalore Universitare NënaTerezë, Tirana, Albania; 4 American Hospital Kosova, Pristina, Kosovo

**Keywords:** Breast cancer, Nottingham Prognostic Index, Immunohistochemistry

## Abstract

**Background:**

Prognosis and treatment of patients with breast carcinoma of no special type (NST) is dependent on a few established parameters, such as tumor size, histological grade, lymph node stage, expression of estrogen receptor, progesterone receptor, and HER-2/neu, and proliferation index. The original Nottingham Prognostic Index (NPI) employs a three-tiered classification system that stratifies patients with breast cancer into good, moderate, and poor prognostic groups. The aim of our study was to use robust immunohistochemical methodology for determination of ER, PR, HER-2/neu, Ki-67, p53, and Bcl-2, and to observe differences in the expression of these markers when patients are stratified according to the original, three-tiered Nottingham Prognostic Index.

**Methods:**

Paraffin blocks from 120 patients diagnosed with breast carcinoma, NST, were retrieved from our archive. Cases included in the study were female patients previously treated with modified radical mastectomy and axillary dissection.

**Results:**

Our study demonstrates that expression of markers of good prognosis, such as ER, PR, and Bcl-2, is seen with higher frequency in good and moderate NPI groups. In contrast, overexpression of HER-2/neu, a marker of adverse prognosis, is more frequent in moderate and poor NPI groups. High proliferation index, as measured by Ki-67, is seen in moderate and poor NPI groups, whereas low proliferation index is seen in good NPI groups.

**Conclusions:**

These data confirm that the original, three-tiered NPI statistically correlates with the expression of prognostic immunohistochemical markers in breast carcinoma NST.

## Background

The prognosis and treatment of patients with breast carcinoma of no special type (NST) are dependent on a few established parameters, such as tumor size, histological grade, lymph node stage, expression of estrogen receptor (ER), progesterone receptor (PR), overexpression of human epidermal growth factor receptor 2 HER-2), and proliferation index determined by Ki-67 [1-5].

Incorporation of genetic information, as determined by gene expression profiling (GEP) [6-8], is becoming a standard of care for patients with breast cancer [9-14]. However, this methodology is mainly restricted to selected institutions and issubject to issues related to sample processing, data interpretation, reproducibility, validation, feasibility, and cost [2]. These are of critical relevance in considering the need to identify the molecular features of individual tumors in routine practice [2].

The Nottingham Prognostic Index Plus (NPI+) evaluates the expression of ten protein biomarkers by immunohistochemistry in order to stratify patients into seven core classes: class 1 (Luminal A), class 2 (Luminal N), class 3 (Luminal B), class 4 (Basal, p53 altered), class 5 (Basal, p53 normal), class 6 (HER-2/neu positive, ER positive), and class 7 (HER-2/neu positive, ER negative) [1,2]. These classes are comparable to those identified by GEP [1,2].

The origins of this system date from a study in 1992, according to which the Nottingham Prognostic Index (NPI) is a numerical value that is calculated by adding the values of tumor diameter (multiplied by a coefficient of 0.2), histological grade (1 to 3), and lymph node stage (1 to 3) [15]. The original NPI employed a three-tiered classification system distinguishing good, moderate, and poor prognostic groups with cut-off points between the values ≤3.4, 3.4 to 5.4, and >5.4 [15]. Over the years, this system has been modified to a four-to-six-tiered stratification with slight variability in interpretation [2,16,17].

The aim of our study was to use robust immunohistochemical methodology for determination of ER, PRHER-2/neu, Ki-67, p53, and Bcl-2, and to observe differences in expression of these markers when patients are stratified according to the original, three-tiered, N PI.

## Methods

Paraffin blocks from 120 female patients diagnosed with breast carcinoma, NST, were retrieved from our archive. Patients had been treated with modified radical mastectomy and axillary dissection, and staged according to the pathologic tumor-node-metastasis (pTNM) system. Histological grade was determined through the Nottingham grading system, and the NPI was calculated accordingly (Table 1). Cases subjected to neoadjuvant therapy and with multifocal and/or multicentric foci were excluded from the study. Biopsy samples had been fixed in 10% neutral buffered formalin and sectioned in 3- 4micron sections. All biopsy samples were previously evaluated by two independent pathologists. The study is conducted in compliance with the principles of Declaration of Helsinki and approved by the Ethical and Professional Committee.

**Table 1:** The original Nottingham Prognostic Index

NPI*	
**Value**	**Prognosis**
≤3.4	Good
3.41 5.4	Moderate
>5.4	Poor

*NPI = tumor size in cm x 0.2 + histological grade [1- 3] + number of positive lymph nodes [1 = 0 nodes; 2 = 1 3 nodes; 3 > 3 nodes].

### Immunohistochemistry

Antigens were retrieved by placing the slides in target retrieval solution for 45 min at 95 98°C (Table 2). The slides were incubated with the primary antibody for 30 min. The visualization was carried out with dextran polymer conjugated with peroxidase and secondary antibody (EnVision+, DAKO, Denmark, K534011) for 30 min.

**Table 2:** Antibodies, vendor, pretreatment, and dilution

**Antibody**	**Clone**	**Source**	**Pretreatment**	**Dilution**
**ER**	1D5	DAKO	pH 9.0	1:35
**PR**	636	DAKO	pH 9.0	1:50
**HER-2**	HercepTest	DAKO	pH 6.1	Ready to use
**Ki-67**	MIB-1	DAKO	pH 9.0	1:100
**p53**	DO-7	DAKO	pH 9.0	1:1000
**Bcl-2**	124	DAKO	pH 9.0	1:100

### Interpretation of results

The interpretation of ER, PR, and HER-2/neu immunohistochemistry was carried out according to American Society of Clinical Oncology (ASCO) guidelines [18,19]. A positive result for ER and PR was any nuclear stain observed in more than 1% of the tumor cells [18]. Non-neoplastic epithelium of the normal terminal duct-lobular unit was used as an internal control. The staining pattern for HER-2/neu was scored on a scale of 0 to 3+. Negative for HER-2/neu overexpression were scores 0 and 1+. Positive results were membranous staining with a score of 3+ [19]. Cases with “equivocal” results (2+) were excluded in order to restrict to immunohistochemical methodology.

The interpretation of proliferative index as measured by Ki-67 was carried out by estimating the percentage of cells with nuclear stain in the most mitotically active areas. The cut-off point between low and high proliferative index was 14%, according to St. Gallen criteria [20].

Cytoplasmic staining with Bcl-2 was interpreted by using a cut-off value of 10% between negative and positive results [21].

The interpretation of p53 was carried out by estimating the proportion of tumor cells with nuclear stain. A positive result was any value above 10% [22].

The data were statistically analyzed with the chi-square and Kruskal-Wallis methods.

## Results

**Figure 1:**
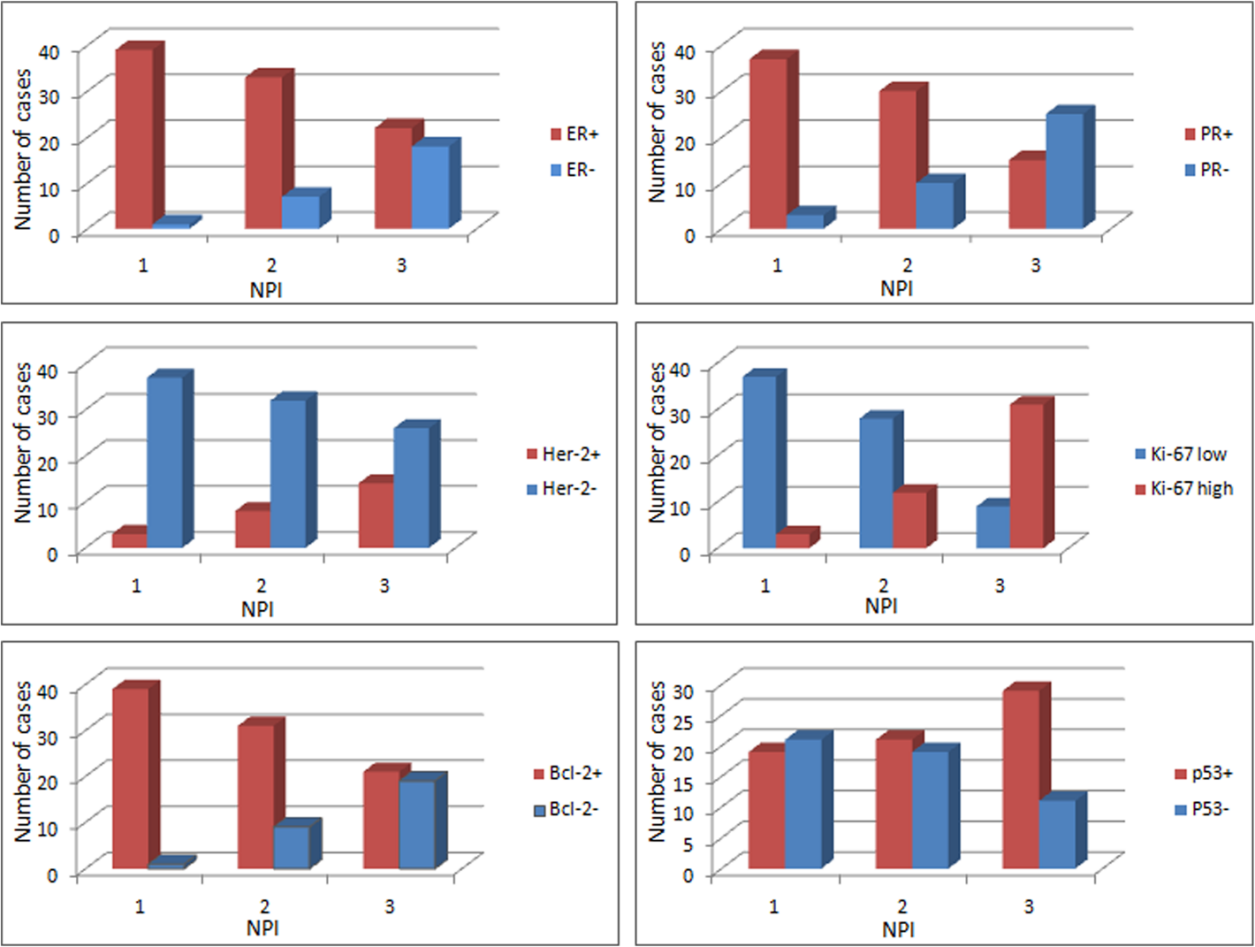
Expression of ER, PR, HER-2/neu, Ki-67, Bcl-2, and p53 in NPI groups.

Paraffin blocks from 120 female patients diagnosed with breast carcinoma, NST, were analyzed. Positive expression of ER and PR was inversely related to the NPI numerical value. This difference was statistically significant between the NPI groups *P* < 0.01); see Figure 1 and Table 3.

**Table 3:** Immunohistochemical marker expression in respective NPI groups

**Marker**	**NPI 1**		**NPI 2**		**NPI 3**		***P*** **value [Kruskal-Wallis]**
	***Neg.***	***Pos.***	***Neg.***	***Pos.***	***Neg.***	***Pos.***	
ER	1	39	7	33	18	22	*P* < 0.01
	2.50%	97.50%	17.50%	82.50%	45.00%	55.00%	
PR	3	37	10	30	25	15	*P* < 0.01
	7.50%	92.50%	25%	75%	62.50%	37.50%	
HER-2	37	3	32	8	26	14	*P* = 0.01
	92.50%	7.50%	80%	20%	65%	35%	
Ki-67 low	3	37	12	28	31	9	*P* < 0.01
	7.50%	92.50%	30%	70%	77.50%	22.50%	
Ki-67 high	37	3	28	12	9	31	*P* < 0.01
	92.50%	7.50%	70%	30%	22.50%	77.50%	
Bcl-2	1	39	9	31	19	21	*P* < 0.01
	2.50%	97.50%	22.50%	77.50%	47.50%	52.50%	
p53	21	19	19	21	11	29	*P* = 0.57
	52.50%	47.50%	47.50%	52.50%	27.50%	72.50%	

Overexpression of HER-2/neu was in direct correlation with NPI numerical value. This difference was statistically significant between the NPI groups (*P* = 0.01); see Figure 1 and Table 3. Similarly, high proliferation index, as measured by Ki-67, was more frequent with increasing NPI value, being statistically significant between the groups (*P *< 0.01, Figure 1 and Table 3). In contrast, low proliferation index, as measured by Ki-67, was in inverse correlation with NPI value (*P *< 0.01).

As with ER, expression of Bcl-2 was inversely related to NPI numerical value. This difference was statistically significant (*P *< 0.01), reemphasizing its role as a marker of good prognosis. Expression of antioncogenic protein p53 was heterogeneously distributed in the NPI groups without any statistically significant difference (*P* = 0.057 Figure 1 and Table 3).

## Discussion

There is ample evidence from clinical, morphological, and molecular genetic studies that breast cancer is a heterogeneous disease [1-4,6-8].

The continuous endeavors of breast cancer researchers are the determination of clinical, morphological, molecular, and genetic indicators for accurate prognostic stratification of patients and the determination of individually tailored therapy [1-16].

Many studies have evaluated combinations of different parameters in order to develop a prognostic profile or prognostic index [1-4,6-12,14,15].

It is generally known that prognosis and treatment of patients with breast carcinoma, NST, are dependent on a few established parameters, such as tumor size, histological grade, lymph node stage, expression of estrogen receptor (ER), progesterone receptor (PR), overexpression of human epidermal growth factor receptor 2 HER-2), and proliferation index determined by Ki-67 [1-16].

Current data also support the use of molecular studies for determination of gene expression profile (GEP), but in some situations, integration of clinico-pathological variables with molecular tests, reproducibility, and cost limit their use [1,2].

The Nottingham Prognostic Index Plus (NPI+) evaluates expression of ten protein biomarkers by immunohistochemistry in order to stratify the patients into seven core classes: class 1 (*luminal A*), class 2 (*luminal N*), class 3 (*luminal B*), class 4 (*basal, p53 altered*), class 5 (*basal, p53 normal*), class 6 (*HER-2/neu positive, ER positive),* and class 7 (*HER-2/neu positive, ER negative)*[1,2]*.* These classes are comparable to those identified by GEP [1,2].

The origins of this system date from the study of 1992 [15]. The original NPI employed a three-tiered stratification system distinguishing *good*, *moderate*, and *poor* prognostic groups [15]. This system has been validated through long periods of clinical follow-up and large multinational and multiinstitutional studies [8-14].

Immunohistochemical determination of ER, PR, HER-2/neu, and Ki-67 is part of the basic histopathological procedures of breast cancer diagnosis in most institutions.

Expression of hormone receptors generally entails favorable prognosis and is an indication for hormonal therapy. In contrast, HER-2/neu overexpression is a marker of adverse prognosis and an indication for trastuzumab therapy.

In line with this general knowledge, we observed that expression of ER and PR was more frequent in NPI groups with favorable prognosis. This expression declined significantly with increasing numerical value. HER-2/neu overexpression was in direct correlation with NPI value. As with ER and PR expression, the frequency of HER-2/neu overexpression was statistically different between the three NPI groups.

Proliferation index, as measured by Ki-67, is a proven prognostic marker [20]. In our study, low and highKi-67 indices were in statistical correlation with low and highNPI numerical value. Some studies have reported on the value of Bcl-2 expression as a good prognostic indicator in breast carcinoma [21,23]. The gene of this protein is activated subsequently to the activation of the estrogen receptor gene, and thus is an indication of ER presence [23]. In our study we observed a strong correlation between ER and Bcl-2 expression, and hence an inverse statistical correlation between Bcl-2 and NPI value.

This is also supported in the study by Zhang *et al.*[24], who concluded the following: 1) expression of Bcl-2 is associated with better response to hormonal therapy, and 2) expression of Bcl-2 is a good prognostic marker irrespective of the nodal status.

Some early studies have reported an inverse correlation between expression of Bcl-2 and immunohistochemical detection of EGFR, HER-2/neu, and p53 [25,26]. Alsabeh *et al.*[27] observed that Bcl-2 expression is more common in breast carcinomas with low MIB count.

In our study, expression of antioncogenic protein p53 was heterogeneously distributed in NPI groups without any statistical correlation. Also, expression of this protein was not in correlation with expression of other markers.

There is sufficient evidence to support the role of p53 in breast carcinogenesis despite observations that mutations of this gene are found at lower frequency compared to those in other solid tumors [28]. Studies related to p53 protein regulation have described new transcription products of p53, highlighting alternative molecular mechanisms, besides mutations, through which p53 is deactivated in breast cancer [28]. The molecular analysis of different stages of p53 protein activity may have diagnostic, prognostic, and therapeutic implications in the future [28].

In early stages of the study, we used a four-to-sixtiered NPI stratification. Even though frequency of expression of prognostic markers was in correlation with NPI value, the statistical significance between the groups could not be reached with respective cut-off values. Hence, the original, three-tiered NPI stratification with cut-off points between values ≤3.4, 3.4 5.4, and >5.4 [15] was more appropriate in statistical terms.

## Conclusion

In conclusion, our study demonstrates that expression of ER, PR, and Bcl-2 is seen with higher frequency in good and moderate NPI groups. In contrast, overexpression of HER-2/neu is more frequent in moderate and poor NPI groups. The Ki-67 proliferation index is in direct correlation with NPI value.

These data confirm that the original, three-tiered Nottingham Prognostic Index statistically correlates with expression of prognostic immunohistochemical markers in breast carcinoma, NST.

### Consent

Written informed consent was obtained from the patients for the publication of this report and any accompanying images.

## Competing interests

The authors declare that they have no competing interests.

## Authors’ contribution

FK designed the study and wrote the manuscript. LGL contributed in writing the manuscript. SHK analyzed data and contributed in writing the manuscript. MA analyzed data. UG carried out statistical analysis and provided important contribution in data interpretation and study design. All authors read and approved the final manuscript.
